# Editorial: Celebrating one year of published articles in the Journal of Extracellular Biology

**DOI:** 10.1002/jex2.87

**Published:** 2023-04-24

The Journal of Extracellular Biology (J Ex Bio) was launched in October 2021 and published its first paper in March, 2022. J Ex Bio is a sister journal to the Journal of Extracellular Vesicles (JEV) and is the second journal published by the International Society for Extracellular Vesicles (ISEV). We are pleased to celebrate the anniversary of the first publication in the journal and the subsequent papers published during the last 12 months. Over the past year, we have published a diverse range of articles on various topics related to extracellular vesicle/particle biogenesis, interactions with each other and with other extracellular macromolecules, the extracellular matrix, and the effects of these vesicles/particles on recipient cells and organs. Articles have covered reviews, short communications, full articles, technical reports and commentaries.

In the first year we have published 40 articles, with more than 32,000 views. The success of J Ex Bio is reflected in the growing interest and importance of extracellular biology in the scientific community. J Ex Bio provides a platform for researchers to share their findings, advance our understanding of this complex field and pave the way for future discoveries.

The first year of J Ex Bio was enabled by the contributions made by our authors, reviewers and editorial board members. We have received numerous high‐quality submissions from researchers worldwide, which have undergone rigorous peer review to ensure that only the most impactful and novel findings are published. Our acceptance rate is just over 61%.

Over the next year, we are committed to maintaining the high standards of our journal and providing a forum for researchers to disseminate their work to a broad audience. We welcome submissions from all areas of extracellular biology and encourage authors to consider publishing their work in J Ex Bio.

To celebrate the first year of publications, we proudly announce the launch of a virtual issue called “One Year in J Ex Bio.” This virtual issue, available on the J Ex Bio website, contains all published material from the first 12 months of the journal.

Finally, we would like to thank our readers for their support and interest in J Ex Bio. We look forward to another successful year of publishing cutting‐edge research in this exciting field.

Andrew F. Hill

Editor in Chief, Journal of Extracellular Biology

